# Poly[(μ_2_-3,6-di-4-pyridyl-1,2,4,5-tetra­zine)(μ_2_-thio­cyanato)copper(I)]

**DOI:** 10.1107/S1600536810001431

**Published:** 2010-01-30

**Authors:** Yiming Wu, Qinglong Meng, Chi Zhang

**Affiliations:** a School of Chemistry and Chemical Engineering, Jiangsu University, 301 Xuefu Road, Zhenjiang 212013, People’s Republic of China; b Institute of Science and Technology, Jiangsu University, 301 Xuefu Road, Zhenjiang 212013, People’s Republic of China

## Abstract

The title compound, [Cu(NCS)(C_12_H_8_N_6_)]_*n*_, is a self-assembled two-dimensional metal–organic network. The Cu atom is linked by two N atoms from two 3,6-di-4-pyridyl-1,2,4,5-tetra­zine ligands and by the N and S atoms from two thio­cyanate ligands in a distorted tetra­hedral environment. The Cu atom and the thio­cyanate ligand occupy a crystallographic mirror plane *m*, and a crystallographic inversion centre is in the middle of the tetra­zine ring, generating the zigzag fashion of the two-dimensional network. The infinite –Cu—SCN—Cu—SCN– chain is due to translational symmetry along the *a* axis. These chains are further connected through the 3,6-di-4-pyridyl-1,2,4,5-tetra­zine ligands that bridge the Cu^I^ centers, generating a two-dimensional network. There are π—π stacking inter­actions between the pyridine and tetra­zine rings (perpendicular distances of 3.357 and 3.418 Å), with a centroid–centroid distance of 3.6785 (16) Å.

## Related literature

For compounds with related architectures, see: Oxtoby *et al.* (2003 ▶); Dinolfo *et al.* (2004 ▶); Hsu *et al.* (2006 ▶); Xue *et al.* (2008 ▶); Withersby *et al.* (1997 ▶, 2000 ▶).
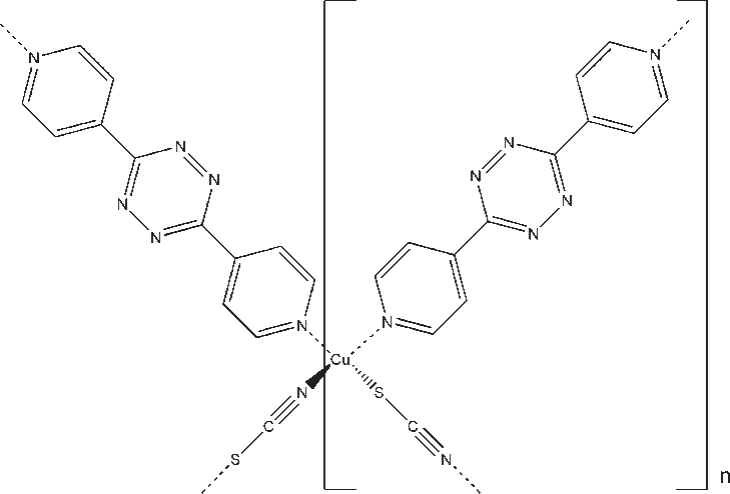


## Experimental

### 

#### Crystal data

[Cu(NCS)(C_12_H_8_N_6_)]
*M* *_r_* = 357.88
Monoclinic, 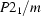

*a* = 5.8640 (12) Å
*b* = 18.510 (4) Å
*c* = 6.3993 (13) Å
β = 104.42 (3)°
*V* = 672.7 (2) Å^3^
*Z* = 2
Mo *K*α radiation
μ = 1.79 mm^−1^
*T* = 250 K
0.20 × 0.20 × 0.20 mm

#### Data collection

Rigaku Mercury diffractometer
Absorption correction: multi-scan (*ABSCOR*; Higashi, 1995 ▶) *T*_min_ = 0.445, *T*_max_ = 0.738
3288 measured reflections
1373 independent reflections
1304 reflections with *I* > 2σ(*I*)
*R*_int_ = 0.018

#### Refinement

*R*[*F* ^2^ > 2σ(*F* ^2^)] = 0.033
*wR*(*F* ^2^) = 0.069
*S* = 1.11
1373 reflections
106 parameters
H-atom parameters constrained
Δρ_max_ = 0.48 e Å^−3^
Δρ_min_ = −0.36 e Å^−3^

### 

Data collection: *CrystalClear* (Rigaku, 2008 ▶); cell refinement: *CrystalClear*; data reduction: *CrystalClear*; program(s) used to solve structure: *SHELXS97* (Sheldrick, 2008 ▶); program(s) used to refine structure: *SHELXL97* (Sheldrick, 2008 ▶); molecular graphics: *SHELXTL* (Sheldrick, 2008 ▶); software used to prepare material for publication: *SHELXL97*.

## Supplementary Material

Crystal structure: contains datablocks I, global. DOI: 10.1107/S1600536810001431/si2230sup1.cif

Structure factors: contains datablocks I. DOI: 10.1107/S1600536810001431/si2230Isup2.hkl

Additional supplementary materials:  crystallographic information; 3D view; checkCIF report

## Figures and Tables

**Table 1:** Selected bond lengths (Å)

Cu1—N2^i^	1.948 (3)
Cu1—N1^ii^	2.100 (2)
Cu1—N1	2.100 (2)
Cu1—S1	2.2550 (13)

Symmetry codes: (i) 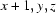; (ii) 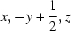.

**Fig. 1.**
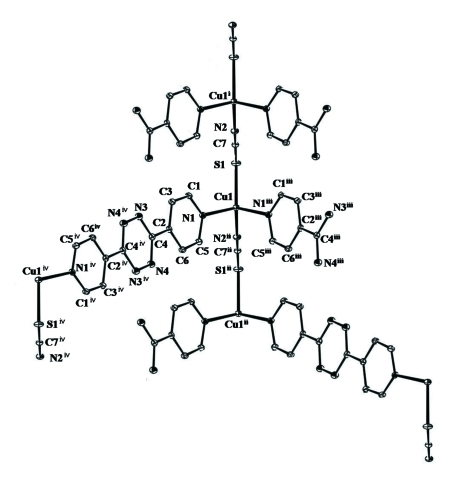
A section of the two-dimensional structure of the title complex, with atom labels and 30% probability displacement ellipsoids. H atoms have been omitted. [symmetry codes: i = -1 + x, y, z; ii = 1 + x, y, z; iii = x, 1/2 - y, z; iv = -1 - x, -y, 1 - z.]

**Fig. 2.**
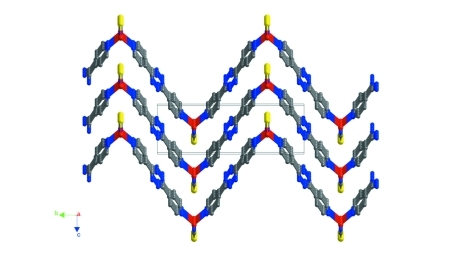
The unit cell packing diagram.

## supplementary crystallographic information

### Comment

The coordination polymers that based on metal halides and N-donor ligands are one of the most important and promising fields in magnetism, nonlinear optics, electronics, catalysis and molecular topologies (Oxtoby *et al.*, 2003; Hsu *et al.*, 2006), the title compound is an example.

Many different coordination modes' polymers can be obtained by 3,6-di-4-pyridyl-1,2,4,5-tetrazine ligands because it can connect two different metal cations, and the Cu atom in CuSCN can be linked by linear spacer ligands into sheets (Dinolfo *et al.*, 2004; Hsu *et al.*, 2006). We obtained a two-dimensional metal-organic compound as the title complex, C_13_H_8_CuN_7_S (Fig. 1), whose structure contains single [CuSCN] ribbons as a characteristic motif. The asymmetric unit of the title compound consist of a half 3,6-di-4-pyridyl-1,2,4,5-tetrazine ligand, half a copper(I) and one SCN . group. Each Cu atom connected by three N atoms and one S atom, give rise to a distorted tetrahedron (Table 1). The layers can be described as formed by two types of perpendicular zigzag like chains crossing at the Cu^I^ centers. Chains of the first type run along the *b*-axis and have 3,6-di-4-pyridyl-1,2,4,5-tetrazine as a bridging ligand, while the second type extend along the *a*-axis containing bridging thiocyanate ligands. The structure is stabilized through π—π stacking interactions which can be imagined in Figure 2, with perpendicular distances between pyridine and tetrazine rings of 3.357 Å [symmetry code for Cg2_tetrazine_: x, y, -1 + z), and of 3.418 Å [symmetry code for Cg1_pyridine_: x, y, 1 + z); the Cg1···Cg2 distance is 3.6785 (16) Å. Cg1 and Cg2 are the centroids of rings (N1, C1, C3, C2, C6, C5) and (N3, C4, N4, N3^iv^, C4^iv^, N4^iv^), symmetry code iv = -1 - x, -y, 1 - z.

### Experimental

CuSCN (12.2 mg) and NH_4_SCN(1.4 mg) were added into 2.5 ml DMF and the solution were stirred for 10 min at room temperature until became clarification. Then, the resulting solution was subsequently filterated to a tube, then 2.5 ml solution of *i.*-pron and 3,6-di-4-pyridyl-1,2,4,5-tetrazine added to afford a black filtrate. Many prismatic black crystals were obtained a few weeks later.

### Refinement

H atoms were positioned geometrically and refined as a riding model, with *U*_iso_(H) = 1.2*U*_eq_ (pyridyl C atoms). The C—H bond lengths are 0.93 Å.

### Crystal data

**Table:**

[Cu(NCS)(C_12_H_8_N_6_)]	*F*(000) = 360
*M**_r_* = 357.88	*D*_x_ = 1.767 Mg m^−^^3^
Monoclinic, *P*2_1_/*m*	Mo *K*α radiation, λ = 0.71073 Å
Hall symbol: -P 2yb	Cell parameters from 2914 reflections
*a* = 5.8640 (12) Å	θ = 3.3–28.9°
*b* = 18.510 (4) Å	µ = 1.79 mm^−^^1^
*c* = 6.3993 (13) Å	*T* = 250 K
β = 104.42 (3)°	Prism, black
*V* = 672.7 (2) Å^3^	0.20 × 0.20 × 0.20 mm
*Z* = 2	

### Data collection

**Table:**

Rigaku Mercury diffractometer	1373 independent reflections
Radiation source: fine-focus sealed tube	1304 reflections with *I* > 2σ(*I*)
graphite	*R*_int_ = 0.018
Detector resolution: 28.5714 pixels mm^-1^	θ_max_ = 26.4°, θ_min_ = 3.3°
dtprofit.ref scans	*h* = −7→5
Absorption correction: multi-scan (*ABSCOR*; Higashi, 1995)	*k* = −22→20
*T*_min_ = 0.445, *T*_max_ = 0.738	*l* = −8→7
3288 measured reflections	

### Refinement

**Table:**

Refinement on *F*^2^	Primary atom site location: structure-invariant direct methods
Least-squares matrix: full	Secondary atom site location: difference Fourier map
*R*[*F*^2^ > 2σ(*F*^2^)] = 0.033	Hydrogen site location: inferred from neighbouring sites
*wR*(*F*^2^) = 0.069	H-atom parameters constrained
*S* = 1.11	*w* = 1/[σ^2^(*F*_o_^2^) + (0.0163*P*)^2^ + 0.7211*P*] where *P* = (*F*_o_^2^ + 2*F*_c_^2^)/3
1373 reflections	(Δ/σ)_max_ < 0.001
106 parameters	Δρ_max_ = 0.48 e Å^−^^3^
0 restraints	Δρ_min_ = −0.36 e Å^−^^3^

### Special details

**Table:**

Geometry. All e.s.d.'s (except the e.s.d. in the dihedral angle between two l.s. planes) are estimated using the full covariance matrix. The cell e.s.d.'s are taken into account individually in the estimation of e.s.d.'s in distances, angles and torsion angles; correlations between e.s.d.'s in cell parameters are only used when they are defined by crystal symmetry. An approximate (isotropic) treatment of cell e.s.d.'s is used for estimating e.s.d.'s involving l.s. planes.
Refinement. Refinement of *F*^2^ against ALL reflections. The weighted *R*-factor *wR* and goodness of fit *S* are based on *F*^2^, conventional *R*-factors *R* are based on *F*, with *F* set to zero for negative *F*^2^. The threshold expression of *F*^2^ > σ(*F*^2^) is used only for calculating *R*-factors(gt) *etc*. and is not relevant to the choice of reflections for refinement. *R*-factors based on *F*^2^ are statistically about twice as large as those based on *F*, and *R*- factors based on ALL data will be even larger.

### Fractional atomic coordinates and isotropic or equivalent isotropic displacement parameters (Å^2^)

**Table:**

	*x*	*y*	*z*	*U*_iso_*/*U*_eq_	
Cu1	−0.50890 (7)	0.2500	−0.44195 (7)	0.03870 (15)	
S1	−0.83431 (16)	0.2500	−0.71812 (15)	0.0502 (3)	
N1	−0.5137 (3)	0.16473 (11)	−0.2265 (3)	0.0379 (5)	
N2	−1.2068 (5)	0.2500	−0.5169 (5)	0.0454 (7)	
N3	−0.7033 (4)	0.00418 (13)	0.3425 (3)	0.0465 (5)	
N4	−0.3006 (4)	0.03426 (13)	0.4828 (3)	0.0467 (5)	
C1	−0.7064 (4)	0.12996 (14)	−0.2042 (4)	0.0407 (6)	
H1A	−0.8441	0.1346	−0.3131	0.049*	
C2	−0.5081 (4)	0.07992 (13)	0.1348 (4)	0.0351 (5)	
C3	−0.7118 (4)	0.08763 (14)	−0.0286 (4)	0.0407 (6)	
H3A	−0.8500	0.0646	−0.0198	0.049*	
C4	−0.5051 (4)	0.03705 (13)	0.3302 (4)	0.0373 (5)	
C5	−0.3166 (5)	0.15430 (15)	−0.0716 (4)	0.0436 (6)	
H5A	−0.1789	0.1761	−0.0871	0.052*	
C6	−0.3058 (4)	0.11312 (14)	0.1092 (4)	0.0417 (6)	
H6A	−0.1643	0.1077	0.2129	0.050*	
C7	−1.0503 (6)	0.2500	−0.5953 (5)	0.0374 (8)	

### Atomic displacement parameters (Å^2^)

**Table:**

	*U*^11^	*U*^22^	*U*^33^	*U*^12^	*U*^13^	*U*^23^
Cu1	0.0309 (2)	0.0500 (3)	0.0350 (2)	0.000	0.00798 (17)	0.000
S1	0.0272 (4)	0.0901 (8)	0.0326 (5)	0.000	0.0059 (3)	0.000
N1	0.0372 (11)	0.0403 (11)	0.0357 (11)	0.0029 (9)	0.0079 (9)	0.0063 (9)
N2	0.0297 (15)	0.057 (2)	0.0506 (19)	0.000	0.0111 (14)	0.000
N3	0.0462 (13)	0.0554 (14)	0.0363 (11)	−0.0009 (11)	0.0070 (9)	0.0117 (10)
N4	0.0458 (13)	0.0558 (14)	0.0369 (11)	−0.0005 (11)	0.0075 (10)	0.0097 (10)
C1	0.0349 (13)	0.0462 (14)	0.0386 (13)	0.0031 (11)	0.0047 (10)	0.0071 (11)
C2	0.0429 (13)	0.0324 (12)	0.0305 (11)	0.0032 (10)	0.0101 (10)	−0.0016 (10)
C3	0.0353 (12)	0.0444 (14)	0.0432 (14)	0.0002 (11)	0.0112 (11)	0.0081 (11)
C4	0.0430 (14)	0.0361 (13)	0.0334 (12)	0.0040 (11)	0.0105 (10)	−0.0004 (10)
C5	0.0376 (13)	0.0484 (15)	0.0426 (14)	−0.0044 (12)	0.0060 (11)	0.0072 (12)
C6	0.0391 (13)	0.0483 (15)	0.0340 (13)	−0.0009 (11)	0.0024 (10)	0.0035 (11)
C7	0.0297 (17)	0.046 (2)	0.0330 (17)	0.000	0.0008 (14)	0.000

### Geometric parameters (Å, °)

**Table:**

Cu1—N2^i^	1.948 (3)	N4—N3^iv^	1.321 (3)
Cu1—N1^ii^	2.100 (2)	N4—C4	1.346 (3)
Cu1—N1	2.100 (2)	C1—C3	1.377 (3)
Cu1—S1	2.2550 (13)	C1—H1A	0.9300
S1—C7	1.648 (4)	C2—C6	1.381 (3)
N1—C5	1.336 (3)	C2—C3	1.385 (3)
N1—C1	1.339 (3)	C2—C4	1.477 (3)
N2—C7	1.150 (4)	C3—H3A	0.9300
N2—Cu1^iii^	1.948 (3)	C5—C6	1.374 (3)
N3—N4^iv^	1.321 (3)	C5—H5A	0.9300
N3—C4	1.332 (3)	C6—H6A	0.9300
			
N2^i^—Cu1—N1^ii^	108.87 (8)	C6—C2—C3	118.0 (2)
N2^i^—Cu1—N1	108.87 (8)	C6—C2—C4	120.7 (2)
N1^ii^—Cu1—N1	97.43 (11)	C3—C2—C4	121.4 (2)
N2^i^—Cu1—S1	116.81 (10)	C1—C3—C2	119.0 (2)
N1^ii^—Cu1—S1	111.55 (6)	C1—C3—H3A	120.5
N1—Cu1—S1	111.55 (6)	C2—C3—H3A	120.5
C7—S1—Cu1	103.11 (12)	N3—C4—N4	125.0 (2)
C5—N1—C1	116.6 (2)	N3—C4—C2	118.0 (2)
C5—N1—Cu1	116.43 (17)	N4—C4—C2	117.1 (2)
C1—N1—Cu1	125.52 (16)	N1—C5—C6	123.7 (2)
C7—N2—Cu1^iii^	168.8 (3)	N1—C5—H5A	118.1
N4^iv^—N3—C4	117.7 (2)	C6—C5—H5A	118.1
N3^iv^—N4—C4	117.3 (2)	C5—C6—C2	119.1 (2)
N1—C1—C3	123.5 (2)	C5—C6—H6A	120.5
N1—C1—H1A	118.2	C2—C6—H6A	120.5
C3—C1—H1A	118.2	N2—C7—S1	177.5 (3)

Symmetry codes: (i) *x*+1, *y*, *z*; (ii) *x*, −*y*+1/2, *z*; (iii) *x*−1, *y*, *z*; (iv) −*x*−1, −*y*, −*z*+1.
